# Nicotine combined with estrogen activates protein kinase PKCι and TAO, while inhibiting specific MAP kinase pathways in cultured human neurons: an atlas of kinase activities for nicotine use disorder

**DOI:** 10.3389/fncel.2026.1807829

**Published:** 2026-06-29

**Authors:** Sally N. Pauss, Zachary A. Kipp, Genesee J. Martinez, Wang-Hsin Lee, Ashley M. White, Cassandra D. Gipson, Terry D. Hinds

**Affiliations:** 1 Drug & Disease Discovery D3 Research Center, Department of Pharmacology and Nutritional Sciences, University of Kentucky College of Medicine, Lexington, KY, United States; 2 Barnstable Brown Diabetes Center, University of Kentucky College of Medicine, Lexington, KY, United States; 3 Markey Cancer Center, University of Kentucky, Lexington, KY, United States

**Keywords:** addiction, PamGene, PamStation, progesterone, serine threonine kinase, smoking, substance use disorder, tyrosine kinase

## Abstract

**Introduction:**

Women exhibit sex-specific differences in their responses to nicotine, with sex hormones like estrogen and progesterone playing key roles in nicotine addiction among women. Nicotine disrupts neuronal firing in the brain’s reward system, an effect regulated by estrogen. In this study, we hypothesized that exposing human female neurons to both nicotine and estrogen would activate distinct signaling pathways.

**Methods:**

We treated human female SH-SY5Y neurons with nicotine and estrogen, and compared these to treatments with each substance alone or vehicle control. Using PamGene PamStation technology, we created an atlas of over 500 kinase activities per sample.

**Results:**

We found that nicotine modulates MAP kinase pathways in a dichotomous manner. Estrogen showed unique kinase effects, and in combination with nicotine, elicited diverse pathway responses—some kinases becoming hyperactive and others hypoactive. Bioinformatics analysis highlighted several kinases as central to this combined signaling, including PKCɩ and TAO, which showed higher kinase activity only with combined treatment and have known links to behavior in rodent models. Conversely, kinases such as the insulin receptor (INSR), HER2, FAK1, and ABL1 exhibited decreased activity under combined treatment.

**Discussion:**

These findings reveal nicotine-specific kinase mechanisms and suggest potential targets for pharmacotherapy aimed particularly at females with high estrogen levels and nicotine use disorder.

## 1 Introduction

Over the last 20 years, epidemiological evidence shows women are more prone to nicotine dependence than men (Giner et al., 2024; Allen et al., 2014). Many studies have explored why women are more vulnerable to nicotine, focusing on sex hormones (Giner et al., 2024; Lynch et al., 2001; Maher et al., 2022), with findings supported by rodent models (Maher et al., 2021). Consequently, the sex steroid hormone estrogen, which binds to activate the estrogen receptor (ER) (Pauss et al., 2025), is considered a key factor in why women often have stronger nicotine cravings (Allen et al., 2014). Women report greater reward from nicotine during the follicular phase, when the estradiol-to-progesterone ratio peaks (Giner et al., 2024; Farris et al., 2019).

Cigarette smoking remains the leading preventable cause of death in the United States, responsible for over 480,000 deaths annually (National Center for Chronic Disease Prevention and Health Promotion (US) Office on Smoking and Health, 2014). Since 2017, there has been an upward trend in nicotine use among middle and high school students (Sun et al., 2021). By 2024, reports show that more than 400,000 middle school students and 1.21 million high school students are e-cigarette users (Park-Lee et al., 2024), with slightly more girls than boys using e-cigarettes (Park-Lee et al., 2024). Also, 22.1% of Americans over age 12 have used tobacco or nicotine products in the past month (SAMHSA, 2024). While tobacco use has declined among men and women of all ages from 2020 to 2022, vaping has risen sharply—by 90% among men aged 18–25 (from 13.4 to 25.4%) and by 125% among women in the same age group (SAMHSA, 2022). Traditionally, men have had higher smoking rates, but this gap may be closing as women’s smoking rates increase more rapidly. This trend suggests a need to explore sex-specific signaling mechanisms related to nicotine use and to develop targeted treatments to support cessation.

Females also experience a telescoping effect, where they may progress into addiction faster than males. This phenomenon has been observed with substances such as alcohol, opiates, and tobacco (Towers et al., 2023). Studies suggest that estrogen may ‘prime’ the reward pathway, influencing this acceleration (Towers et al., 2023; Lynch and Taylor, 2004; Kerstetter et al., 2012). In female rats, ovariectomy (OVX)—the surgical removal of ovaries that causes a sudden drop in female hormones—led to reduced nicotine intake and demand. Notably, daily administration of the estrogen 17β-estradiol (E2) after OVX, which causes a return to a non-cyclic state, increased nicotine consumption but not to the levels seen in ovary-intact females (Maher et al., 2021, 2022; Flores et al., 2016). These findings imply that estrogen plays a role in nicotine use and help explain sex-based differences in nicotine dependence. While women can develop dependence on nicotine more readily than men, they may also experience unique risk profiles. Some studies found that women were more susceptible to lung cancer (Hansen et al., 2018), while others have found that smoking risks are equal (Bain et al., 2004). Nevertheless, women frequently encounter higher relative risks for particular conditions, including heart attack, coronary heart disease, and colorectal neoplasia, even at reduced levels of tobacco consumption (Allen et al., 2014; Prescott et al., 1998).

To better understand how estrogen and nicotine may work together to produce synergistic signaling effects, female neuroblastoma SH-SY5Y neurons were treated with E2 and nicotine combined and individually. Their kinome activity was then analyzed by quantifying more than 500 pathways in real time using the PamGene PamStation microarray-based kinome technology. The PamStation kinase PamChips identified changes in kinase activity induced by each treatment alone and by their combination, revealing the underlying signaling mechanisms of the interaction between estrogen and nicotine. We hypothesized that nicotine alone would regulate growth- and survival-related pathways, as previously demonstrated (Nishioka et al., 2010; Schaal and Chellappan, 2014), whereas estrogen may activate both similar and diverse signaling mechanisms compared to nicotine, including the SRC-p21^RAS^-ERK pathway (Migliaccio et al., 1998). Nicotine signaling involves kinases that bind to nicotinic acetylcholine receptors (nAChRs), thereby triggering calcium influx and activating key pathways, including ERK (mitogen-activated protein kinase 1, *MAPK1*) and CaMKII (calcium/calmodulin-dependent protein kinase II) (Liu et al., 2007; Jia et al., 2021; Zhao et al., 2009). These pathways promote synaptic plasticity, dopamine release, and lasting changes in the brain’s reward circuitry, ultimately leading to nicotine dependence by altering gene expression and cell functions. Kinases such as Protein Kinase A (PKA), PKC, CaMKII, and ERK are central to these adaptations (Liu et al., 2007; Jia et al., 2021; Zhao et al., 2009). We found that the combination of nicotine with estrogen modulates signaling cascades that affect pathways previously published to be associated with behavior and growth; in some respects, they are synergistic, whereas in others, they are antagonistic.

## 2 Materials and methods

### 2.1 Cell culture and treatments

The undifferentiated neuroblastoma cell line, SH-SY5Y, derived from a 4-year-old girl, was seeded in Dulbecco’s Modified Eagle Medium (DMEM) supplemented with 10% fetal bovine serum and 1% antibiotics-antimycotics. The cells were subjected to treatment with a vehicle control, 1 μM E2, 100 μM nicotine, or a combination of E2 and nicotine within their standard media. This cell line exhibits some properties of catecholaminergic neurons, including dopaminergic and adrenergic markers, but does not fully recapitulate those of mature neurons (Xicoy et al., 2017). The combination treatment involved pretreating the cells with nicotine dissolved in water for 30 min, followed by a 6-h exposure to E2 dissolved in 100% ethanol. The nicotine and estrogen alone groups were treated simultaneously (totaling 6.5 h for nicotine and 6 h for estrogen) to match the combination. Subsequently, the cells were harvested concomitantly, and both protein and RNA were extracted.

### 2.2 PamGene PamStation sample preparations

Protein was extracted from the samples utilizing the Mammalian Extraction Reagent (Thermo Fisher Scientific, CAT #78503), Halt Phosphatase Inhibitor (Thermo Fisher Scientific, CAT #78503), and Protease Inhibitor Cocktail (Sigma, CAT #P2714). Protein concentrations were quantified in triplicate using the Pierce BCA Protein Assay Kit (Sigma, CAT #P2714). Subsequently, the samples were diluted to 0.5 μg/μL and combined with the requisite reagents for analysis on the PamStation protein-tyrosine and serine–threonine kinase (PTK and STK) chips. 1 μg of protein per sample was applied to each array for STK analysis, and 5 μg for PTK analysis. The assays were conducted on the PamStation12 platform (PamGene International, ‘s-Hertogenbosch, The Netherlands). Three independent biological replicates per treatment group were processed across three PamChips for both PTK and STK analyses. Kinase activity of 196 PTK and 144 STK substrates was quantified using fluorescently labeled antibodies to detect phosphorylation differences, as we previously described (Lee et al., 2025; Park et al., 2024; Bates et al., 2024; Bates et al., 2023; Badmus et al., 2023; Creeden et al., 2022).

### 2.3 Kinome data bioinformatic analysis

The images captured by the PamStation were exported and analyzed utilizing the Tercen BioNavigator software. Linear regression slopes of signal intensity across cycles and exposure times were used to determine the signal ratio; those that showed nonlinearity were excluded from subsequent analysis. Fold change was computed for each phosphor-peptide based on the average signal ratio across triplicate measurements. Differential phosphorylation was identified using established thresholds of <0.70 and >1.30, indicating a 30% differential signal, which was determined by previous literature (Lee et al., 2025; Park et al., 2024; Bates et al., 2023, 2024; Badmus et al., 2023; Creeden et al., 2022; Lee et al., 2026; Kipp et al., 2026). Upstream kinase identification was conducted using the BioNavigator Upstream Kinase Analysis (UKA) software from PamGene and the Kinome Random Sampling Analyzer (KRSA) package (DePasquale et al., 2021). MEOW plots were generated utilizing KRSA’s [Log2 Fold Change (FC) of kinase substrates * *Δ* confidence (experimental hits/mean hits of 2000 random sampling iterations)] as described in our prior work (Bates et al., 2023). PANDI plots were produced employing the BioNavigator UKA median kinase statistic (MdKS), which indicates changes in kinase activity relative to the control. The kinome phyla trees were created using CORAL, as we previously described (Lee et al., 2025; Bates et al., 2023, 2024).

### 2.4 Cytoscape human brain kinome network analysis

The protein–protein interaction and kinase-substrate database was built from gene matrix transposed (GMT) files downloaded from KEA3 (Kuleshov et al., 2021). Network edges were weighted based on the number of independent source databases supporting each interaction. Any interactions found in only one dataset were removed. These interactions were then filtered to include only genes expressed in the human brain, providing tissue-specific biological context. Human brain gene expression was determined using mRNA expression consensus data from the Human Protein Atlas. Since SH-SY5Y cells are not specific to a single brain region, the maximum expression level across all brain regions was used for each gene. The expression values were log-transformed (log1*p*). A final cutoff (cutoff = mode − 2*SD) was applied to identify brain-expressed genes, as previously described (Creeden et al., 2022). The network-edge database was then filtered to exclude genes with expression below this cutoff. This brain-expressed interaction database was used to generate both network types. The KEA3 substrate network was constructed by inputting significantly changed PTK and STK substrates for each comparison into KEA3 (Kuleshov et al., 2021). This produced a list of enriched kinases (top 10 by MeanRank score) and overlapping proteins, which served as input for the brain-expressed interaction network to generate edges and nodes. The upstream kinase analysis network was assembled using the top and bottom 25 kinases identified from the BioNavigator PTK and STK analysis for each comparison. These kinases were mapped onto the brain-expressed interaction network to generate edges and nodes. Both network types were visualized in Cytoscape (Shannon et al., 2003).

### 2.5 Quantitative real-time PCR analysis

All compounds used for treatment were run in biological triplicate and validated by at least two replicate runs. Total RNA was extracted using a QIAzol Lysis Reagent (Qiagen 79306), chloroform/ethanol separation, and the RNeasy Mini Kit (Qiagen 74106). RNA concentrations were determined with a NanoDrop spectrophotometer (Thermo Fisher Scientific, Wilmington, DE) to prepare for complementary DNA (cDNA) synthesis, which was performed using the cDNA Reverse Transcription Kit (Applied Biosystems). Quantitative real-time PCR was conducted using TrueAmp SYBR Green qPCR SuperMix (Alkali Scientific). The thermocycling protocol consisted of an initial 5-min step at 95 °C, followed by 60 cycles of 15 s at 95 °C, 30 s at 60 °C, and 30 s at 72 °C, culminating in a melt curve analysis from 60 °C to 95 °C. The expression levels of target genes were normalized to the housekeeping gene *36B4*. The primer sequences are listed in Supplementary Table 1.

### 2.6 Statistical analysis

Differences between treatment groups were assessed using a one-way analysis of variance followed by Dunnett’s *post hoc* test. A *p*-value of less than 0.05 was deemed significant. Statistical analyses were conducted with GraphPad Prism 10 (GraphPad Software, Inc., San Diego, CA, USA). Kinase data from the PamStation were initially processed with BioNavigator software, which provides information on substrate phosphorylation and individual kinases. These data were also analyzed using the KRSA (kinase random sampling analysis) package (DePasquale et al., 2021) in R (version 4.5.1), which is suitable for analyzing both single kinases and kinase families. Results from both methods were combined and presented in this manuscript.

## 3 Results

### 3.1 Phosphotyrosine kinase (PTK) signaling analysis

**Figure 1:**
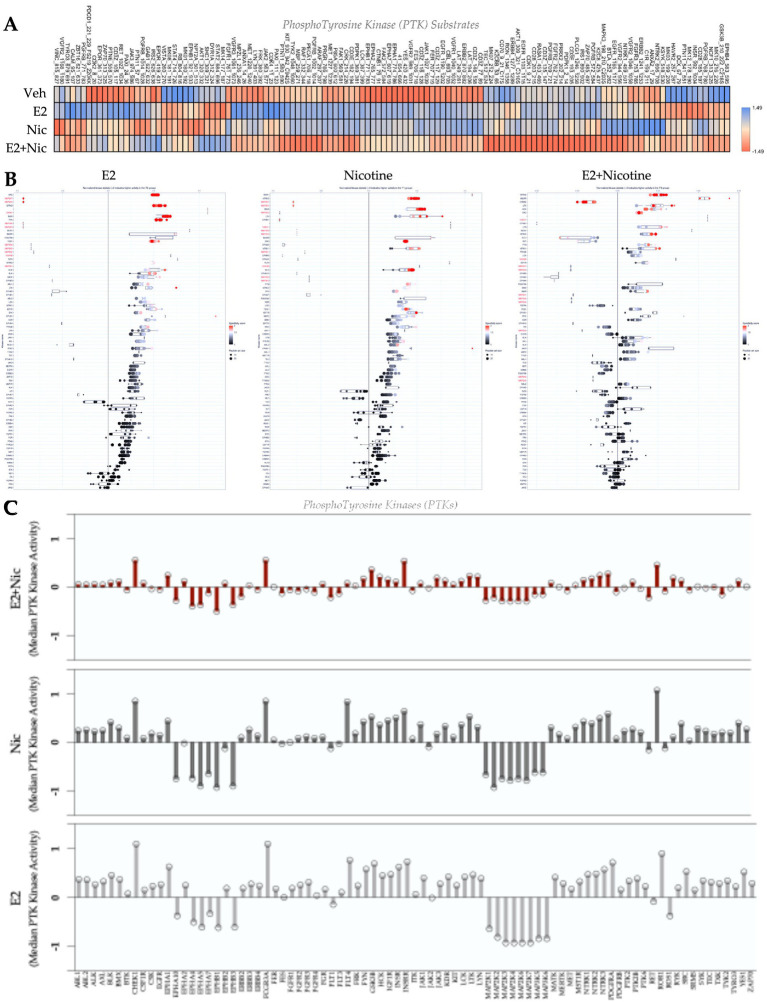
Assessment of phospho-tyrosine kinase (PTK) families. **(A)** Heatmap illustrating the phosphorylation levels of each phosphotyrosine kinase (PTK) substrate across various treatment groups. **(B)** Waterfall plots generated by BioNavigator representing the median kinase statistic for each PTK relative to the vehicle control. These are ranked according to the median kinase statistic. The red coloration in the figures indicates higher specificity, and red text on the kinase names indicates those classified as PTKs and serine–threonine kinases (STKs). **(C)** The median kinase statistics obtained from BioNavigator for each treatment are presented in alphabetical order. Veh, vehicle; E2, 17-estradiol; Nic, nicotine.

Using the PamGene PamStation and its associated software, BioNavigator, along with other bioinformatics tools described below, we determined substrate phosphorylation and quantified kinase activity in our samples. Figure 1A presents a heatmap of mean substrate phosphorylation levels in the PTK PamChip, showing all substrates for each treatment condition. Substrate phosphorylation serves as an indicator of kinase activity, as demonstrated in Figures 1B,C. These figures indicate that the overall PTK response to combination treatment is attenuated relative to either treatment administered individually. Additionally, individual kinases or groups of kinases exhibiting differential responses to the treatments can be readily identified. For instance, HER2 (*ERBB2*) demonstrated increased activity in both the E2 and nicotine single treatments but showed decreased activity in the combination treatment. FGFR1, FGFR2, and FGFR3 showed modest increases with individual treatments, but their activity was reduced in the combination treatment. Table 1 lists the top changed kinases for each treatment identified by BioNavigator with the median kinase statistic, which reflects the magnitude of change.

**Table 1:** Top changed PTK kinases compared to vehicle.

E2	MdKS	Nicotine	MdKS	E2 + nicotine	MdKS
CHEK1	1.091	ROR1	1.075	CHEK1	0.558
FCGR3A	1.091	EPHB1	−0.930	FCGR3A	0.558
MAP2K3	−0.933	MAP2K2	−0.929	INSRR	0.538
MAP2K6	−0.933	EPHA5	−0.899	EPHB1	−0.498
MAP2K4	−0.930	EPHB3	−0.899	ROR1	0.459
MAP2K7	−0.930	CHEK1	0.850	EPHA4	−0.391
ROR1	0.895	FCGR3A	0.850	EPHA5	−0.365
MAP3K5	−0.846	FLT4	0.838	EPHB3	−0.365
MAP3K6	−0.846	MAP2K4	−0.786	GSK3B	0.360
MAP2K2	−0.817	MAP2K7	−0.786	MAP2K4	−0.289
FLT4	0.761	EPHA10	−0.750	MAP2K7	−0.289
INSRR	0.730	MAP2K3	−0.748	MAP2K3	−0.285
PDGFRA	0.710	MAP2K6	−0.748	MAP2K6	−0.285
GSK3B	0.687	EPHA4	−0.739	PDGFRA	0.277
MAP2K1	−0.645	MAP2K1	−0.667	EPHA10	−0.275

The table displays the top 15 most changed PTK kinases as measured by the BioNavigator median kinase statistic for each treatment compared to the control. Veh, vehicle; E2, 17β-estradiol; Nic, nicotine; MdKS, median kinase statistic.

**Figure 2:**
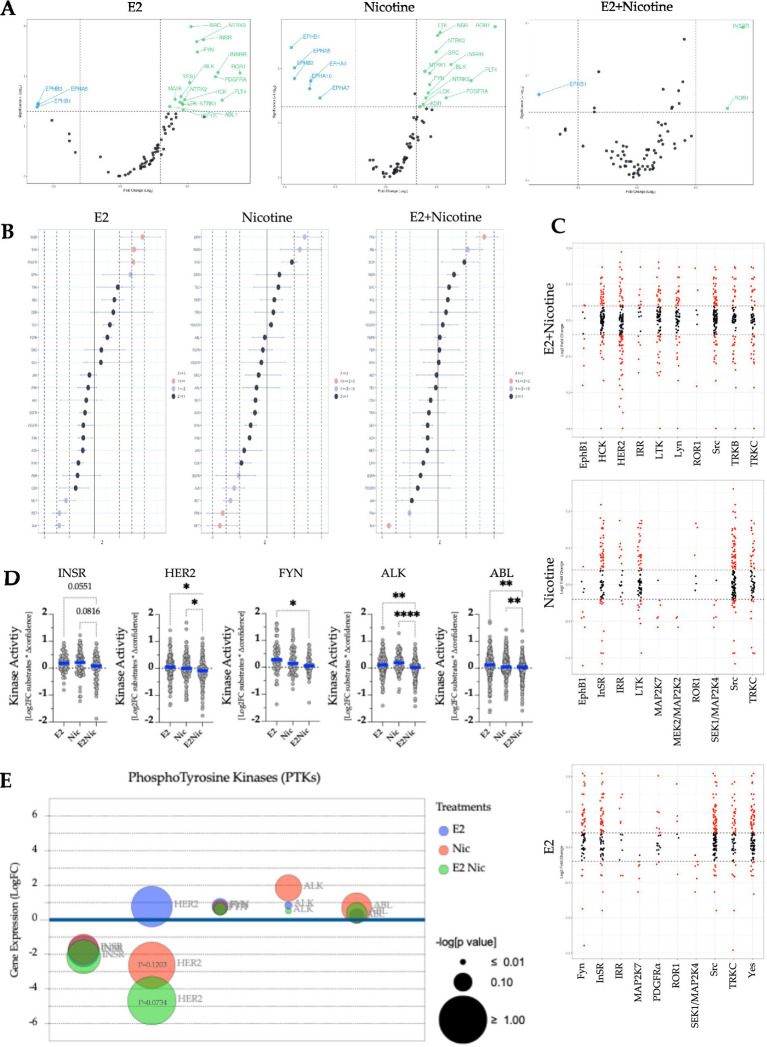
Evaluation of the phospho-tyrosine kinase (PTK) individual pathways. **(A)** Volcano plots generated by BioNavigator software display the Log2 fold change of kinase responses for each treatment relative to the control. Significantly decreased kinases are depicted in blue, whereas significantly increased kinases are shown in green. The dotted lines indicate the thresholds for statistical significance. **(B)** *Z*-score plots, produced by KRSA, rank kinase families based on kinase activity for each treatment relative to the control. **(C)** Reverse KRSA plots, also generated by KRSA, illustrate the top 10 most altered kinases in the treatment compared to the control. These plots represent the Log2 fold change in substrate phosphorylation relative to the control for all substrates of the top kinases. The dotted line signifies the fold change cutoff. **(D)** MEOW plots to visualize kinase activity, as measured by substrate phosphorylation, along with the confidence levels for the kinases of interest, where ^*^p < 0.05, ^**^p < 0.01, and ^****^p < 0.0001. **(E)** mRNA expression levels were quantified using real-time quantitative PCR for genes of interest. The location of each circle corresponds to the average fold change in expression relative to the control, and the circle size is the −log(*p*-value) [*p*-values that were significant or close to significance were written within the circle]. Veh, vehicle; E2, 17-estradiol; Nic, nicotine.

These initial analyses help us identify the upstream kinases that phosphorylate substrates in signaling pathways. To further elucidate the upstream kinases, Figure 2A presents volcano plots of kinase activity relative to the vehicle control, highlighting those with statistically significant differences. In both individual treatments with E2 or nicotine, numerous kinases exhibit significant alterations; in the combined treatment, only three kinases are notably affected: ROR1 and INSRR show increased activity, whereas EPHB1 demonstrates decreased activity. Figure 2B displays the *Z*-score of kinase family activity compared to the control in order to delineate the most hyperactive and hypoactive kinases. Figure 2C categorizes the top ten most altered kinases identified by the Kinome Random Sampling Analyzer (KRSA) package to generate Reverse KRSA plots, which illustrate substrate phosphorylation for all substrates associated with a particular kinase (DePasquale et al., 2021). E2 treatment results in elevated kinase activities, including FYN, INSR, SRC, and YES, as evidenced by increased phosphorylation levels of their substrates. Nicotine treatment increases the activity of INSR, IRR, LTK, ROR1, SRC, and TRKC, while decreasing the activity of EPHB1, MAP2K7, MEK2/MAP2K2, and SEK1/MAP2K4. The most significantly affected kinases under combined E2 and nicotine treatment largely overlap with those altered by either treatment alone. Utilizing substrate phosphorylation and KRSA confidence scores, MEOW plots were generated to illustrate the activity levels of specific kinases. HER2, FYN, ALK, and ABL kinase activities were suppressed by combining E2 with nicotine, as indicated by MEOW plot analysis (see Figure 2D). The mRNA expression levels of the most altered PTK kinases were validated across various bioinformatics analyses, as depicted in Figure 2E. Interestingly, HER2, FYN, ALK, and ABL mRNA expression was all higher in E2 combined with nicotine treatments compared to the vehicle. With kinase activity low and expression elevated, indicating possible negative feedback of their signaling mechanisms.

### 3.2 Serine–threonine kinase (STK) analysis

**Figure 3:**
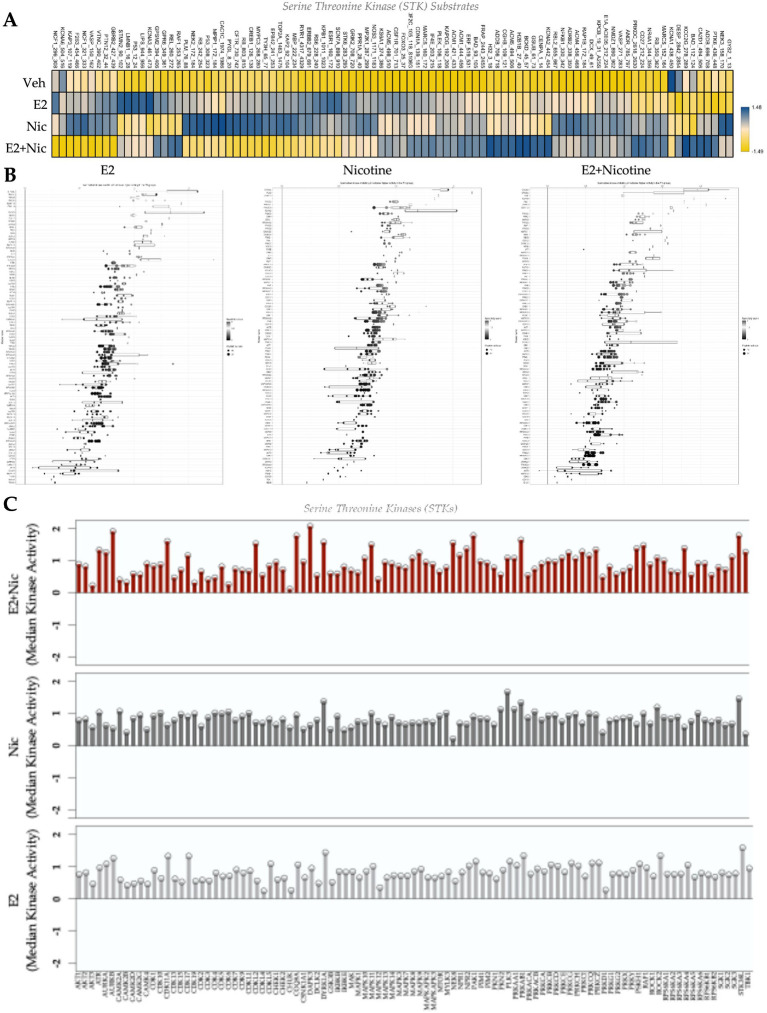
Evaluation of serine–threonine kinase (STK) families. **(A)** A heatmap depicting the phosphorylation levels of each serine–threonine kinase (STK) substrate across various treatment groups. **(B)** Waterfall plots generated using BioNavigator, illustrating the median kinase statistic for each protein tyrosine kinase (STK) within the respective treatment relative to the control, arranged by median kinase statistic. **(C)** The median kinase statistic for each treatment, derived from BioNavigator, is presented in alphabetical order. Veh, vehicle; E2, 17-estradiol; Nic, nicotine.

**Figure 4:**
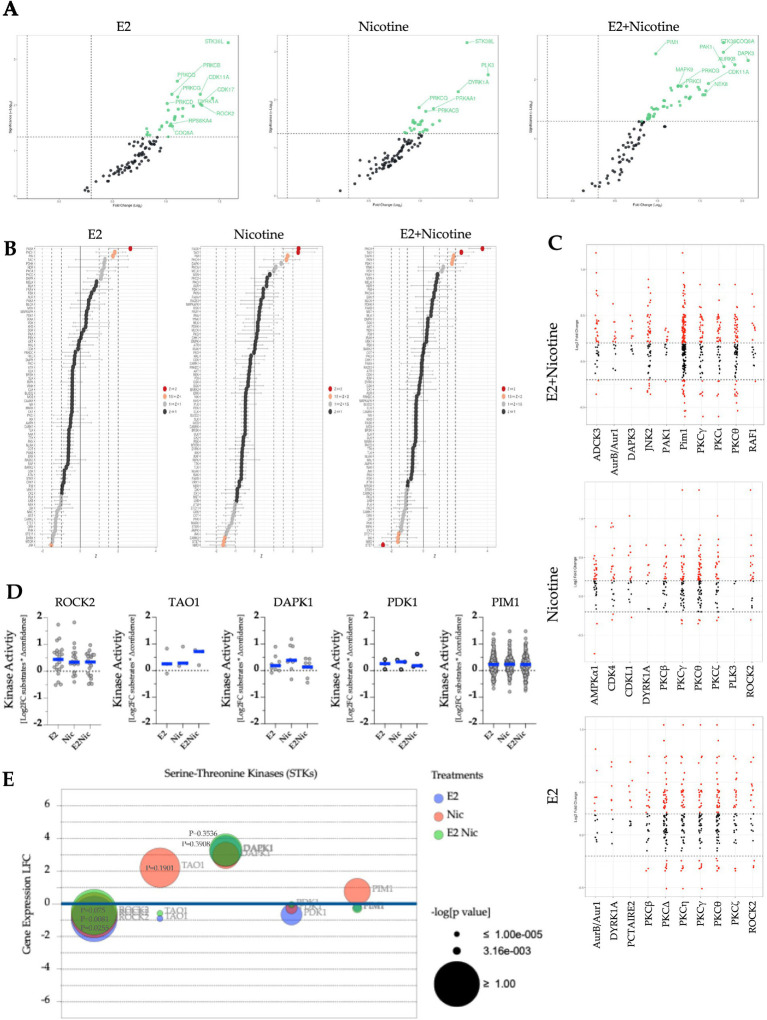
Serine–threonine kinase (STK) individual pathway assessment. **(A)** Volcano plots generated by BioNavigator depict the Log2 fold change in kinase activity within a treatment relative to the control. Significantly elevated kinases are highlighted in green. The dotted lines indicate the significance thresholds. **(B)** *Z*-score plots, created by KRSA, rank kinase families by kinase activity for each treatment relative to the control. **(C)** Reverse KRSA plots, also generated by KRSA, show the top 10 kinases with the largest changes in the treatment relative to the control. The Log2 fold change in substrate phosphorylation, compared to its phosphorylation in the control, is presented for all substrates of these top kinases. The fold change cutoff is indicated by the dotted line. **(D)** MEOW plots illustrate kinase activity, measured by substrate phosphorylation, along with the confidence levels for the kinases of interest; ^*^p < 0.05. **(E)** mRNA expression levels were quantified using real-time qPCR for selected genes. The location of each circle corresponds to the average fold change in expression relative to the control, and the circle size is the −log(*p*-value) [*p*-values that were significant or close to significance were written within the circle]. Veh, vehicle; E2, 17-estradiol; Nic, nicotine.

We conducted an analogous analysis of the STK PamChip data using the same procedure as for the PTK PamChip. Generally, the STK substrates exhibited elevated phosphorylation responses across all treatments compared with the vehicle control (Figure 3A). The kinase activity is further illustrated in PANDI plots and through Table 2, with all STK kinases demonstrating heightened activity relative to the control (Figures 3B,C). Table 2 lists the most changed STK kinases and their activity scores for each treatment. Unlike PTK kinases, the combined treatment resulted in STK hyperactivity surpassing that observed with either treatment alone. Nevertheless, this pattern was not consistent across all STKs; for example, AKT3 and CDK6 showed diminished activity when both treatments were combined compared to the individual treatments. The kinases ROCK2, PIM1, TAO1, DAPK1, and PDK1 were notably affected by the combination therapy relative to single treatments, as corroborated by all analytical tools. UKA quantification suggested that most STKs were hyperactive, as illustrated in Figure 4A. *Z*-score analysis identified the most hyper- and hypo-active STK kinases (Figure 4B). The ten most significantly altered kinases, as identified by the KRSA package for generating reverse KRSA plots, are categorized in Figure 4C; these plots depict substrate phosphorylation associated with each kinase. The MEOW plot shown in Figure 4D indicates that TAO (Thousand And One Amino Acid Protein Kinases) was raised and potentially hyperactive only under the combined E2 and nicotine treatment conditions. However, substrate data for TAO kinase analysis are limited, which constrains the analysis. The mRNA expression levels of the most significantly altered STK kinases were validated, as depicted in Figure 4E. TAO mRNA expression was increased with nicotine but suppressed with E2 alone and the combination of E2 with nicotine.

**Table 2:** Top changed STK kinases compared to vehicle.

E2	MdKS	Nicotine	MdKS	E2 + nicotine	MdKS
STK38L	1.583	PLK3	1.667	DAPK3	2.071
DYRK1A	1.436	STK38L	1.460	AURKB	1.914
ROCK2	1.337	DYRK1A	1.374	PAK1	1.784
PRKAB1	1.335	PRKAB1	1.338	STK38L	1.781
CDK17	1.322	ROCK2	1.191	COQ8A	1.778
CDK11A	1.321	PRKAA1	1.132	PRKAB1	1.646
AURKB	1.256	PKN2	1.124	CDK11A	1.602
PAK1	1.158	CAMK2A	1.069	DYRK1A	1.571
PLK3	1.155	PRKACB	1.056	NEK8	1.560
PRKCZ	1.115	CDK6	1.050	CDKL2	1.535
PRKCG	1.110	ATR	1.029	MAPK11	1.495
PRKCQ	1.106	CDK4	1.020	RAF1	1.480
CDKL5	1.085	MYLK3	1.015	RPS6KA4	1.388
AURKA	1.081	CDK10	1.013	NPR2	1.376
PSKH1	1.079	CDKL1	1.006	PSKH1	1.375

The table displays the top 15 most changed STK kinases as measured by the BioNavigator median kinase statistic for each treatment compared to the control. Veh, vehicle; E2, 17β-estradiol; Nic, nicotine; MdKS, median kinase statistic.

**Figure 5:**
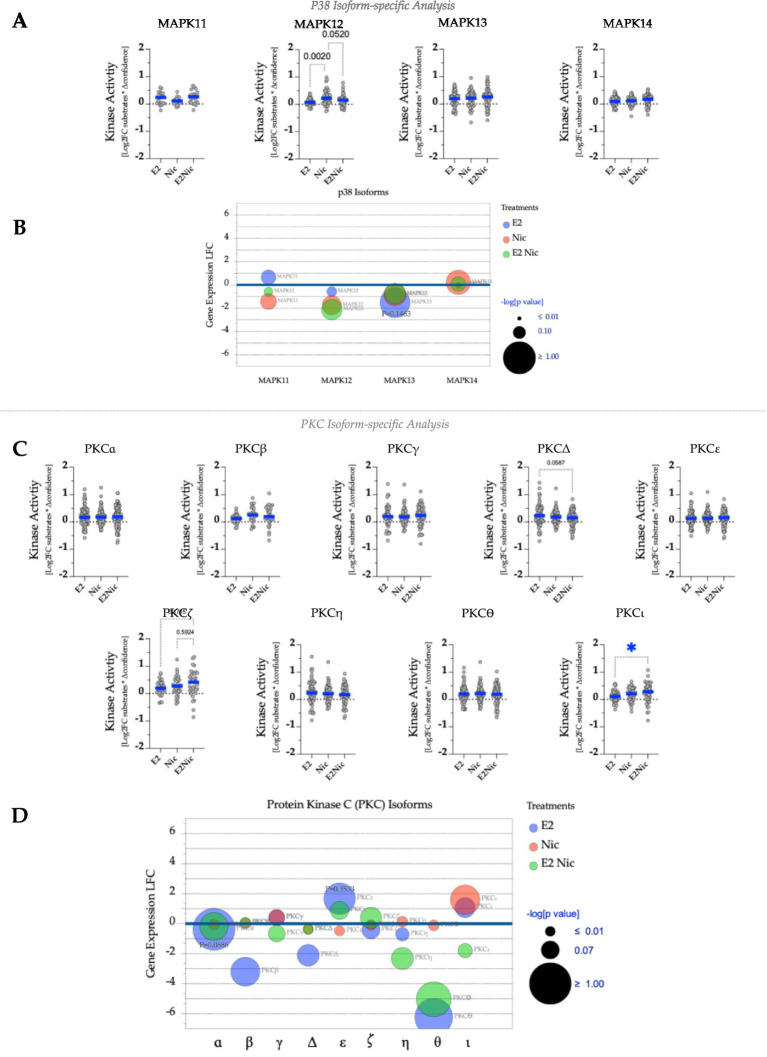
P38 MAPK and protein kinase C (PKC) isoform-specific assessment. **(A)** MEOW plots of P38 isoform-specific kinase activity, measured by substrate phosphorylation, along with the confidence levels for the kinases of interest; ^*^p < 0.05. **(B)** mRNA expression levels were quantified using real-time qPCR for selected genes. The location of each circle corresponds to the average fold change in expression relative to the control, and the circle size is the −log(*p*-value) [*p*-values that were significant or close to significance were written within the circle]. **(C)** MEOW plots of PKC isoform-specific kinase activity, measured by substrate phosphorylation, along with the confidence levels for the kinases of interest; ^*^p < 0.05. **(D)** mRNA expression levels were quantified using real-time qPCR for selected genes. The location of each circle corresponds to the average fold change in expression relative to the control, and the circle size is the −log(*p*-value) [*p*-values that were significant or close to significance were written within the circle]. Veh, vehicle; E2, 17-estradiol; Nic, nicotine.

The analysis specific to PKC isoforms indicated that only atypical PKCι showed a significant difference, with the combination of E2 and nicotine differing across the three conditions (Figure 5C). While the activities of several PKC isoforms were changed with all three compounds, the mRNA expression of the PKC isoforms indicated that they were not significantly changed (Figure 5D). Meanwhile, PKCΔ decreased, though not statistically significant (*p* = 0.0587), and PKC𝜁 increased, also not statistically significant (*p* = 0.5924). Isoforms of PKC, including the atypical PKCs, are implicated in neuronal plasticity and, together with MAPKs such as TAO, have been shown to mediate rewarding behaviors and responses to drugs like nicotine and cocaine (Lee and Messing, 2011; Isotani et al., 2025; Jin et al., 2005; King et al., 2011; Kapfhamer et al., 2013). The activity of the p38 isoforms within the MAPK family showed some differences in kinase activity. MAPK12 had significantly higher activity with nicotine treatment (Figure 5A). However, these differences were not reflected in the gene expression (Figure 5B). TAO protein kinases regulate signaling pathways from carbachol to P38 MAP kinases (P38 isoforms are MAPKs11-14) and ternary complex factors (Chen et al., 2003), which may account for the alterations observed in several MAP kinases.

### 3.3 Full kinome overview represented by phyla trees and networks

**Figure 6:**
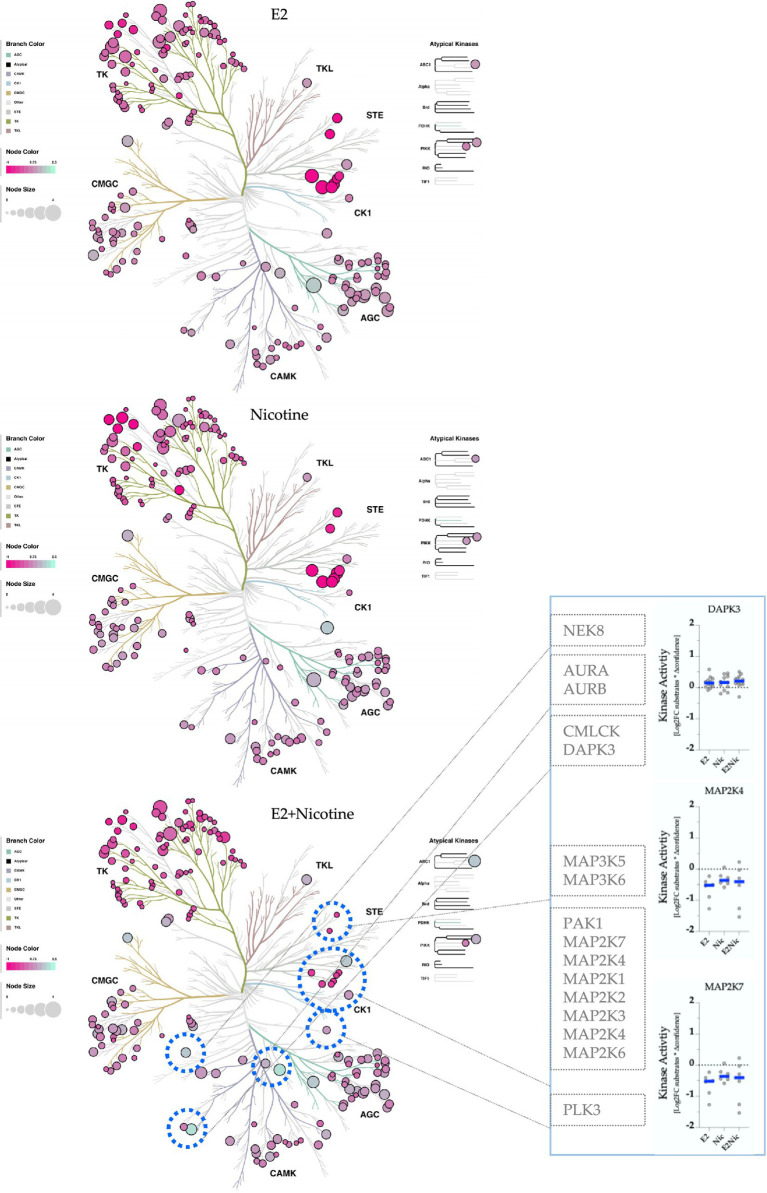
Comparisons of kinase phyla. Phylogenetic trees illustrate variations in PTK and STK kinase activity for each treatment relative to the control. The color of the nodes signifies kinase activity based on the median kinase statistic, whereas node size denotes the level of statistical significance. Kinases of particular interest are highlighted with blue circles.

**Figure 7:**
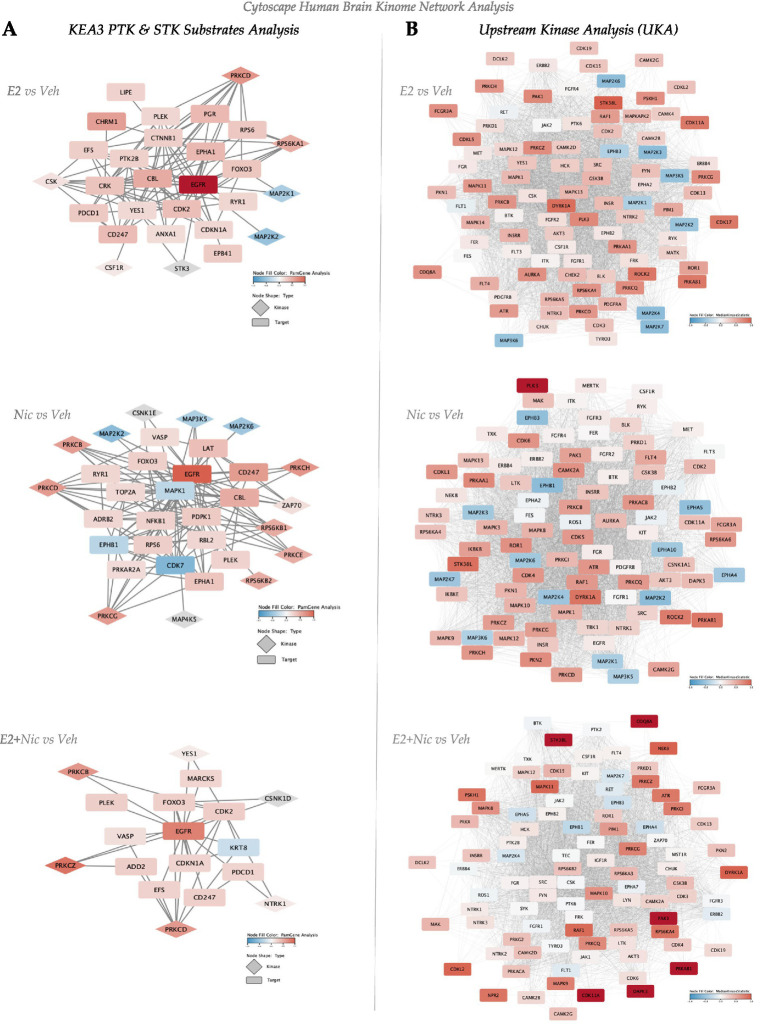
Cytoscape human brain kinome network analysis. **(A)** Network of KEA3 PTK and STK substrate analysis for each treatment compared to control, visualized using Cytoscape. The differentially phosphorylated substrates (squares, colored by log2 fold change) for each comparison are mapped to upstream kinases (diamonds, colored by median kinase statistic; gray indicates implicated kinases through KEA3 but not BioNavigator UKA). The network includes only interactions between proteins with known expression in the human brain. **(B)** Network of the top and bottom 25 PTK and STK kinases identified by BioNavigator UKA, showing brain-expressed interactions. The node color indicates the median kinase statistic for each comparison to control.

The STK and PTK kinase data were organized into phylogenetic trees to illustrate how each treatment influenced kinase activity, grouped by family (Figure 6), or into networks to show the connectivity of substrates and upstream kinases (Figure 7). The color of each node indicates the median kinase statistic, which reflects kinase activity levels. The size of the nodes represents the final score, indicating the confidence level in the observed change in activity. Blue circles highlight individual kinases or clusters of kinases that exhibited differences in the combination treatment relative to either treatment alone. The NEK8 node in the combination treatment is distinguished by a different color and a larger size than the nodes within the individual treatment trees, signifying its hyperactivity and the high confidence associated with this finding in the combination. Several MAP kinases are notably suppressed. Overall, the phyla trees indicate that the combination of E2 with nicotine induces signaling mechanisms that are dichotomous and divergent.

## 4 Discussion

The findings in this study enhance our understanding of how estrogen and nicotine interact in human neuronal cells, providing more detailed insights into their intracellular signaling pathways. Higher estrogen levels have been linked to increased nicotine reward in women, especially during the follicular phase when reinforcement perceptions are at their peak. However, the specific signaling mechanisms involved in the estrogen-nicotine interaction, such as the activation of PKCι and TAO kinases and the inhibition of various MAP kinases, have not been extensively studied.

Some experts view addiction as a maladaptive learning disorder where drug-induced changes in the brain, such as remodeling of the prefrontal cortex, lead to difficulties in memory formation, decision-making, and reward-seeking behavior (DePoy and Gourley, 2015; Rezayof et al., 2023). Reward-related learning involves communication among the basal ganglia (including the nucleus accumbens), the limbic system (including the hippocampus), and the prefrontal cortex (Rezayof et al., 2023). In this system, the nucleus accumbens, rich in dopaminergic neurons, mediates pleasure and reinforcement via dopamine surges. The hippocampus plays a key role in memory formation and retrieval, creating strong associations between the drug and its effects. The prefrontal cortex contributes to decision-making and impulse control, which can be compromised by drug use. The olfactory bulb also has a role, especially in nicotine dependence, as smells trigger memories and cravings (Cortese et al., 2015). These memories influence behavior through synaptic plasticity, where cellular structure, gene expression, and function undergo long-lasting modifications in response to stimuli. Long-term potentiation and depression are forms of increased or decreased sensitivity to stimuli, involving persistent changes in protein expression that modify neuronal reactivity (Rezayof et al., 2023). Additionally, neurogenesis and dendritic growth, critical aspects of synaptic plasticity in addiction, are discussed further below.

While this study primarily examined the mechanistic interactions between nicotine and E2 in human neurons, it is important to note that many members of the MAPK/ERK and PKC kinase families are implicated in reward behaviors (Jia et al., 2021; Satoh et al., 2011; Trainor et al., 2010; Bennison et al., 2020; Engel et al., 2009). For example, knocking down CAMK2 in mice prevented nicotine-induced conditioned place preference, a typical sign of addiction (Jackson et al., 2016). Additionally, ERK activity and phosphorylation levels rose in the nucleus accumbens, prefrontal cortex, and hippocampus after nicotine and other addictive substances (Jia et al., 2021). PKCs play a crucial role in long-term potentiation, which is vital for memory and habit formation; thus, they are relevant to reward and reinforcement (Park-Lee et al., 2024). These kinases are found in several brain regions, including the olfactory bulb and hippocampus, both of which are linked to addiction (Olive and Messing, 2004). The role of the PKC family in addiction has been thoroughly researched (Olive and Messing, 2004); for instance, PKCι-deficient mice showed increased ethanol consumption and reduced pharmacological responses (Olive and Messing, 2004). PKCs are activated via phosphorylation, commencing with activation of PDK1 (Olive and Messing, 2004). Our results indicate that PDK1 activity increased with either estrogen or nicotine alone, but the increase was smaller when they were combined.

Other kinases, such as TAO1, which participate in neuronal cell proliferation and microtubule formation (Fang et al., 2020), showed evidence of hyperactive signaling in this combination. However, more work is needed to make this determination. Similar to other members of the TAO family, TAO1 has been strongly associated with various neurodevelopmental behavioral disorders, including autism (SAMHSA, 2024; SAMHSA, 2022), and exhibits dysregulation in neurological disorders (Byeon, 2024). A study employing the same cellular model utilized in our research, SH-SY5Y neurons, demonstrated that TAO1 activation induces apoptosis and is regulated by JNK (c-Jun N-terminal kinase) signaling (Towers et al., 2023). This finding is corroborated by our data, which indicated that both TAO1 and JNK2 were hyperactive following treatment with E2 and nicotine. In addition to the JNK stress response pathway, the TAO family also regulates the p38 pathway (Fang et al., 2020). Both of these stress response pathways have also been implicated in addiction (El Rawas et al., 2020; Bruchas and Chavkin, 2010). Studies in Drosophila have shown that TAO plays a role in the behavioral response to ethanol, cocaine, and nicotine (Byeon, 2024). TAO kinases also regulate the cytoskeleton and neuritogenesis, with TAO1 inducing microtubule instability and TAO2 stabilizing them (Fang et al., 2020). Neuroplasticity and synaptogenesis are essential to addiction formation, and cytoskeletal growth plays an integral role in both processes (DePoy and Gourley, 2015). For example, nicotine has been shown in rodent models to induce dendritic spine proliferation in the prefrontal cortex (DePoy and Gourley, 2015; Brown and Kolb, 2001). Overall, TAO’s induction by E2 and nicotine may contribute to nicotine dependence via the induction of stress response and cytoskeletal pathways.

The PamStation uniquely facilitates the concurrent evaluation of multiple kinase activities. The mRNA levels of these kinases do not entirely reflect their functional activity, underscoring the importance of this technology. For example, *INSR* mRNA expression is reduced under the combination treatment relative to E2 treatment, a finding supported by the observed kinase activity. HER2 kinase activity was significantly suppressed with the combination treatment and increased with E2 alone. Conversely, *HER2* mRNA expression increased with the combination treatment despite a decline in its activity. A complex aspect of the PamStation technology is the need for upstream pathway deconvolution, which relies on databases. As noted, we primarily rely on two software packages: BioNavigator and KRSA, both of which use multiple databases. Because they use different database collections, they do not share the same set of kinases, which partially accounts for differences in the top kinases identified by the two software tools. Using both software allows us to expand the number of kinases for which we can obtain data and provides validation when both software reach the same conclusions. Regarding HER2, both kinase analysis software packages demonstrated concordance, identifying it as a significantly affected kinase in the combination treatment. The kinases ALK, FAK1, ABL, and INSR all exhibited diminished responses to the combination treatment.

Nicotine pretreatment reduces estrogen-driven PTK kinase activity but increases STK kinase activity. The activities of STK’s PKCι and TAO1 were increased when combining E2 with nicotine, and both kinases have supporting literature that implicates them in nicotine’s rewarding behaviors. PKCι, an atypical protein kinase, is essential for survival signaling, especially via NNK (Nitrosamine 4-Methylnitrosamino-1-(3-pyridyl)-1-butanone), a potent carcinogen in tobacco smoke that supports lung cancer cell survival (Shen et al., 2012). NNK activates PKCι and FAK1, promoting cancer cell migration, invasion, and wound healing (Shen et al., 2012). Additionally, NNK inhibits BAD phosphorylation, a pro-apoptotic protein, thereby preventing apoptosis and promoting cell survival.

While these kinases are likely involved in behavioral responses, it is important to consider other contributing factors, such as TAO, which are involved in the development of the central nervous system (CNS) (King et al., 2011; Byeon, 2024). TAO kinases are distinguished by a highly conserved serine/threonine kinase domain that modulates MAP signaling pathways (Chen and Cobb, 2001; Hutchison et al., 1998; Tassi et al., 1999; Yasuda et al., 2007). Beyond their catalytic activity, TAO kinases also influence cytoskeletal organization through interactions with actin and tubulin, mediated by a structurally distinct C-terminal tail region (Johne et al., 2008; Mitsopoulos et al., 2003; Moore et al., 2000; Timm et al., 2003; Zihni et al., 2006). The mechanisms of TAO signaling are complex and are likely involved in drug-dependent behaviors; however, their role in neuronal development warrants greater concern. This is particularly relevant given the increasing prevalence of e-cigarette use containing nicotine among adolescents. Estrogen levels peak in both females and males around ages 10–12 (Igarashi et al., 2021; Zec et al., 2012; Frederiksen et al., 2020). Males typically exhibit E2 levels of approximately 150 pmol/L at peak, whereas females tend to have levels of approximately 1,600 pmol/L (Frederiksen et al., 2020). These findings indicate that further studies on TAO and its role in nicotine-associated behaviors are needed to better elucidate its physiological and signaling functions, especially given that other sex hormones may also influence these signaling mechanisms.

Progesterone and its metabolite allopregnanolone activate the progesterone receptor (PR) activity (Pauss et al., 2025) and enhance GABA-A receptor signaling (Kapur and Joshi, 2021; Guennoun et al., 2015; Kaura et al., 2007). This effect reduces mesolimbic dopaminergic activity and stress-related circuitry, which are linked to lower nicotine craving, withdrawal symptoms, and cue reactivity, depending on sex and phase (Lynch and Sofuoglu, 2010; Novick et al., 2022b; Wetherill et al., 2021). Analyses comparing nicotine with or without progesterone in male and female neurons, including PR antagonism and GABA-A–specific measures, reveal model- and sex-specific mechanisms (Novick et al., 2022a,b; Gunn et al., 2011). Future research should include male neurons to explore potential sex differences. A detailed kinome analysis of androgens and nicotine in male and female neurons might clarify the reasons for observed sex differences. Moreover, the androgen receptor (AR) interacts with stress hormone receptors (McBeth et al., 2016; McBeth et al., 2015; Martinez et al., 2024) and could play a role in mediating neurosignaling pathways involved in addiction.

The limitations of this study include the use of 100 μM nicotine and 1 μM E2, which exceed typical plasma levels in smokers (0.1–1 μM) and in women (about 0.1–1.6 nM) (Frederiksen et al., 2020). A report on e-cigarette users found plasma nicotine at 17.9 ng/mL (110.3 nM) (Hiler et al., 2017). Esther et al. (2023) reported airway nicotine levels in active smokers ranging from 70 to 850 ng/mL (roughly 0.5 to 5 μM), which remained elevated even after 24 h of abstinence (Hiler et al., 2017). The 30-min nicotine pretreatment followed by 6 h of E2 exposure may not reflect long-term exposure related to addiction. For E2, concentrations ranging from 0.01 to 10 μM are standard in SH-SY5Y cells to reliably activate the ER and downstream pathways, overcoming issues such as serum binding, metabolism, and membrane permeability that limit the use of lower doses (Ding et al., 2019). SH-SY5Y neurons tolerate up to 10 μM E2 in short-term experiments (Ding et al., 2019), allowing clear ER-dependent gene expression, neurite growth, and neuroprotection without significant toxicity under controlled conditions. Yang et al. (2010) showed that 10 μM E2 provides estrogen-mediated neuroprotection in the rat hippocampus. Another limitation is that the kinome data are descriptive and lack functional validation of key kinases. It remains uncertain whether kinase inhibitors targeting these pathways alter neuronal responses to nicotine and estrogen *in vivo*. Claims of behavioral relevance are speculative without such studies, and reward experiments in rodents are necessary.

In summary, the combination of nicotine and estrogen triggers kinase signaling pathways that might affect behavioral outcomes. The functions of PKCι, TAO, and other MAP kinases in nicotine addiction remain unclear. However, the increasing use of e-cigarettes among young people raises concerns, as TAO and related signaling molecules are involved in neuronal development and may impact cognitive functions over time. This research presents a kinome atlas that can serve as a foundation for future studies of these kinases and their roles in the addiction cycle. Future investigations should include signaling pathways during withdrawal and relapse. Overall, further research is essential to fully understand their role in reward-related behavior in both rodent and human studies. Additionally, future research should investigate molecular differences between sexes and why women might be more susceptible to nicotine dependence.

## Data Availability

The original contributions presented in the study are publicly available. This data can be found here: 10.6084/m9.figshare.32619816.
