# Supplementary material for: Nicotine combined with estrogen activates protein kinase PKCι and TAO, while inhibiting specific MAP kinase pathways in cultured human neurons: an atlas of kinase activities for nicotine use disorder
Source: Front Cell Neurosci. 2026 Jun 29;20:1807829. doi: 10.3389/fncel.2026.1807829 (PMC13356939; doi:10.3389/fncel.2026.1807829)
Supplement: Supplementary file 1 [file Table_1.DOCX]

Supplementary Material

**Supplementary Table 1:** Sequences of the real-time PCR primers.

| Gene | Forward Primer | Reverse Primer |
| --- | --- | --- |
| *INSR* | CTATTACCGCAAGGGTGGGA | CAGAGTACCACGCCAAAGGA |
| *HER2* | CTCCATTGGGACCGGAGAAA | ATGGTGCTCACTGCGGC |
| *FYN* | AGAGCATCAGCAAGAGTAGCA | GATGGCGCAACTGCAACG |
| *ALK* | GAGGGGGCGGCAAGATTTC | GTTTGCAGCGTCCTTGCTC |
| *ABL1* | GTGGGCTGCAAATCCAAGAA | ATGCTACTGGCCGCTGAAG |
| *ROCK2* | GACCTGAGCGCGAGGAG | TAAGCCATCCAGCAAGCTCTC |
| *TAO1* | CTAGAGCTGGAATGCCGTCG | ATTGTGTTGAGGTGGCGGAA |
| *DAPK1* | CACTCCCTAGCTGTGTTCCC | CTTCCCAGACCATCACCACC |
| *PDK1* | TGCTGTATGGCCTGCAAGAT | ACATTCTGGCTGGTGACAGG |
| *PIM1* | GATTTCCGACTGGGGAGAGC | TAATGACGCCGGAGAAACCC |
| *MAPK11* | CAAGGCCAGCAGCCATATCT | TCAAGGCATCAGGAACCGAG |
| *MAPK12* | ACATGAGAAGCTAGGCGAGG | TCAGGTCTCTGTGGATGATGC |
| *MAPK13* | ACAAGCAGGACGTCAACAAGA | ACAAGCAGGACGTCAACAAGA |
| *MAPK14* | AGGATGCCAAGCCATGAGG | TCGCAAAGTTCATCTTCGGC |
| *PRKCA* | ACGAGGTGAAGGACCACAAA | AAACTTGGCACTGGAAGCCT |
| *PRKCB* | GACCTGAAGGCGAACGTGAT | TCTCTTGTCTCTAGCTTTTGGCT |
| *PRKCD* | CTGGTGGTTGGTGCGTTGT | ATAGGAGTTGAAGGCGATGCG |
| *PRKCE* | GCTCGTCTTCTCTTCTGGAGG | TGTATTTCCTCGCCGGGTTC |
| *PRKCG* | CCTACGTGAACCCCGACTTC | CTGGGGTGCAGGATATGACG |
| *PRKCH* | TGAACTTGGAACCTGGCGGT | GCGAGCTTCAAACCAGGAAG |
| *PRKCI* | CACACTTTCCAAGCCAAGCG | GGCGTCCAAGTCCCCATATT |
| *PRKCQ* | ACCCATTCTCAGCAGACTCC | GCACGAGAAGGGGTTAAGGT |
| *PRKCZ* | TTCGAGGAGCTCTGTGAGGAA | CTTCGCTGTCCACCCACTTG |
